# The transcriptional landscape of insect galls: psyllid (Hemiptera) gall formation in Hawaiian *Metrosideros polymorpha* (Myrtaceae)

**DOI:** 10.1186/s12864-015-2109-9

**Published:** 2015-11-16

**Authors:** Sebastian Bailey, Diana M. Percy, Charles A. Hefer, Quentin C. B. Cronk

**Affiliations:** School of Biological and Chemical Sciences, Queen Mary University of London, London, E1 4NS UK; Department of Life Sciences, Natural History Museum, London, SW7 5BD UK; Agricultural Research Council, Private Bag X05, Onderstepoort, Pretoria, 0110 South Africa; Department of Botany, University of British Columbia, 3529-6270 University Blvd., Vancouver, BC Canada V6T 1Z4; Jodrell Laboratory, Royal Botanic Gardens, Kew, Richmond, Surrey TW9 3AB UK

**Keywords:** Gall evolution, Plant development, Gene ontology, Auxin, Gall morphology, Psyllidae, Plant-insect interactions

## Abstract

**Background:**

Recent studies show that galling Hymenoptera and Diptera are able to synthesize the plant hormone indole-3-acetic acid (auxin) from tryptophan and that plant response to insect-produced auxin is implicated in gall formation. We examined the leaf transcriptome of galled and ungalled leaves of individuals of the Hawaiian endemic plant *Metrosideros polymorpha* (Myrtaceae) subject to infestation by psyllid (Hemiptera) gall-makers in the genus *Trioza* (Triozidae).

**Results:**

Transcript libraries were sequenced using Illumina technology and the reads assembled *de novo* into contigs. Functional identification of contigs followed a two-step procedure, first identifying contigs by comparison to the completely sequenced genome of the related *Eucalyptus*, followed by identifying the equivalent *Arabidopsis* gene using a pre-computed mapping between *Eucalyptus* and *Arabidopsis* genes. This allowed us to use the rich functional annotation of the *Arabidopsis* genome to assess the transcriptional landscape of galling in *Metrosideros*. Comparing galled and ungalled leaves, we find a highly significant enrichment of expressed genes with a gene ontology (GO) annotation to auxin response in the former. One gene consistently expressed in all galled trees examined but not detected in any libraries from ungalled leaves was the *Metrosideros* version of SMALL AUXIN UPREGULATED (SAUR) 67 which appears to be a marker for leaf-galling in *Metrosideros*.

**Conclusions:**

We conclude that an auxin response is involved in galling by *Metrosideros* psyllids. The possibility should therefore be considered that psyllids (like other insects examined) are able to synthesize auxin.

## Background

### The biology of galls - what is known?

A gall is a plant structure resulting from the alteration of plant developmental processes by a galling organism, and which increases the fitness of the galler by providing a nutrient rich, protected environment [1]. It can be considered an extension of the phenotype of the galling organism [2]. Galls are caused by a variety of organisms but notably bacteria, nematodes, insects, mites and fungi. Most gallers are parasites, but a few, like the pollinating wasps of figs (*Ficus*) are beneficial to the host. Many of the parasitic galling organisms, such as the gall midge pests of cereals [3, 4], are of considerable economic importance as serious agricultural threats. Despite this importance remarkably little is known about the mechanisms by which the developmental processes of the plant host are perturbed by the parasite. However it has been established that at least some insect gallers directly synthesize the plant growth regulator auxin, specifically the major auxin indole-3-acetic acid (IAA) [5–7]. Auxin induces expression of two major classes of auxin-responsive genes: the GH3 family and the small auxin upregulated (SAUR) family [8]. A study of galling by the Asian rice gall midge (Rawat et al. 2012) found induction of both GH3 and SAUR genes [9].

A diverse array of insects form galls, particularly in the Hymenoptera, Diptera and Hemiptera [10]. In the Hemiptera, gall-making is particularly abundant in the aphids [11, 12], scale insects [13] and the psyllids [14–17]. The subject of this paper is a radiation of psyllid gall-makers on the common native Hawaiian tree, *Metrosideros polymorpha*.

### *Metrosideros* in Hawaii – its morphology and its galls

*Metrosideros* is a tree and shrub genus of the eucalyptus and guava family, Myrtaceae, with around 50 species distributed across the Pacific region and a small radiation in the Hawaiian Islands [18–20]. One species, *M. polymorpha* (local name: ‘ōhi’ lehua) is an abundant, variable and ecologically important species in the Hawaiian Islands [21, 22]. It is host to a group of galling psyllids, which are often so abundant that they disfigure the leaves throughout the plant [23]. *M. polymorpha* has glabrous (hairless) biotypes as well as hairy-leaved biotypes and the hairy-leaved biotypes are noticeably less heavily galled than the hairless. No complete genome of *Metrosideros* has so far been released but good genomic resources are available for *Eucalyptus* [24–27], which, like *Metrosideros*, is in the family Myrtaceae.

### The gall-makers – a radiation of species and gall types

Psyllids, or jumping plant lice, form the superfamily Psylloidea (Hemiptera, suborder Sternorhyncha). They are plant phloem feeders, usually highly host-specific and several are important agricultural pests such as the potato psyllid (*Bactericera cockerelli*) and the Asian citrus psyllid (*Diaphorina citri*) [28]. The immature stages may be free-living or may form galls of various types from simple pits to more elaborate structures. Because of the economic importance of some psyllids, genomic resources are emerging rapidly [29–32]. A number of endemic psyllids are present on the Hawaiian Islands [23, 33] including a radiation of the genus *Trioza* feeding exclusively on *Metrosideros*. [34]. These psyllid species include stem galling, leaf galling (flat and cone galls), pit galling and free-living types. A modern revision of Hawaiian psyllids, including descriptions of the new species referred to here, is currently being undertaken by one of us (DMP).

**Fig. 1:**
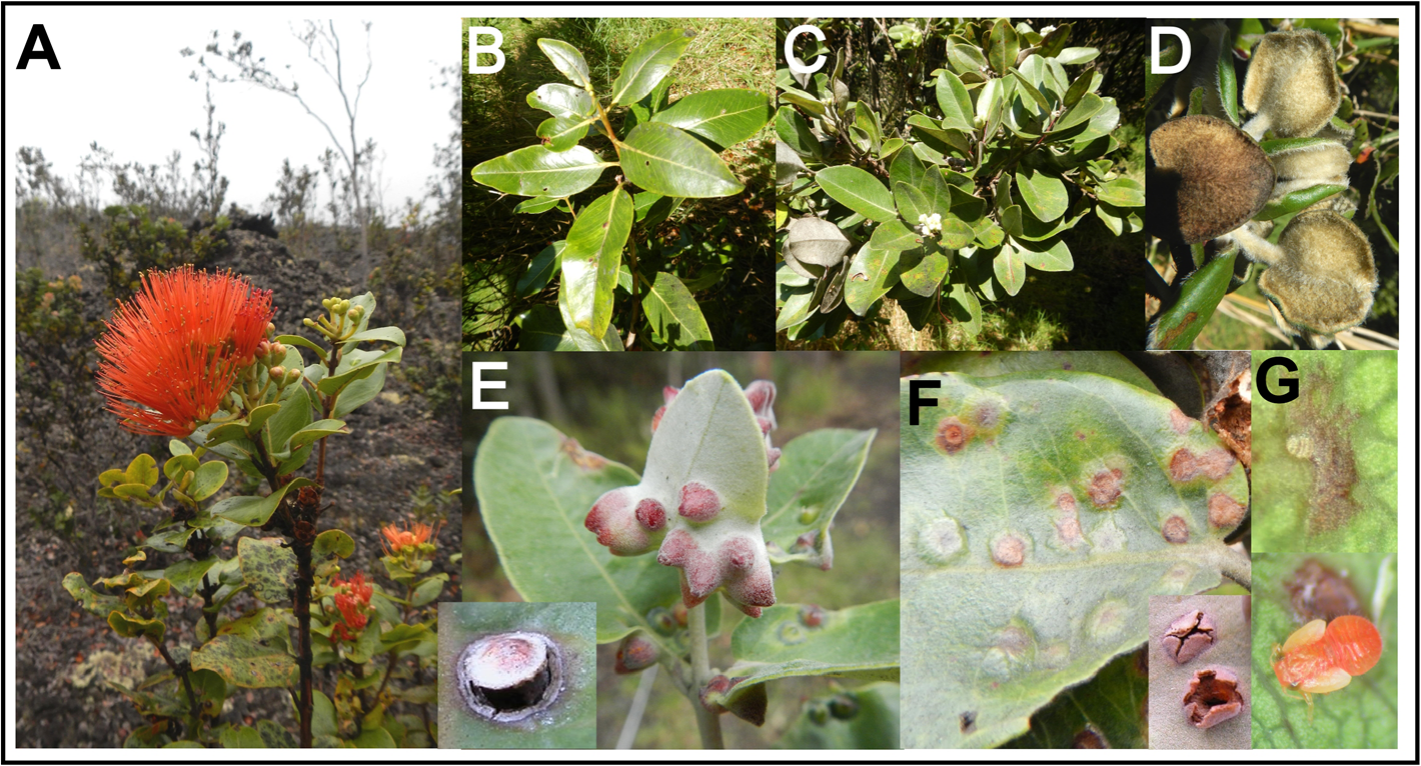
*Metrosideros polymorpha*. Figure 1. **a** View of plant. **b**-**d** Left to right, glabrous, intermediate and pubescent morphs. **e** Cone galls (inset: opening of cone gall on upper surface of leaf). **f** Flat galls (inset: opening of flat gall on lower leaf surface). **g** Psyllid nymphs (*Trioza*), above: first instar before gall formation; below: 5^th^ instar removed from gall

On the island of Hawai’i, two of the leaf gallers (producing flat and cone galls) are common and often gall the same individual leaves. In the early 1990s, a common garden was established on the island of Hawai’i to grow and study different morphotypes of *M. polymorpha* [35, 36], and over time these leaf galling psyllids have colonized many of the plants in the common garden. Both psyllids are native locally and can be found in adjacent forest areas. Cone and flat leaf gallers produce recognizably different gall phenotypes. These galls also dehisce by different mechanisms: flat leaf galls will usually dehisce on the underside of the leaf by irregular fissures, and cone leaf galls dehisce on the upper surface by a circular fissure that gives the appearance of a trap door (Fig. 1). A closely related species on the island of O’ahu, which also produces flat leaf galls, *Trioza ohiacola*, lays its eggs on the lower leaf surface. When the first instar nymph hatches and starts feeding, it is initially exposed on the surface of the leaf; the leaf tissue, which often shows a reddish discolouration at the site of feeding, then encloses the nymph and eclosion to the second instar occurs in a completely closed gall. All instars up to the last (fifth) instar remain in the closed gall (Fig. 1). A similar progression is likely to occur in both psyllid species studied here.

In order to study the perturbation of leaf developmental processes by the leaf-galling species we analyse the transcriptome of galled and ungalled leaves.

## Results

### Insect contigs from galled and ungalled leaves

Contigs from plant 816 (leaf samples with and without galls) were mapped against a transcriptome of an adult Hawaiian psyllid (flat leaf galler, *Trioza nov. sp.* 1). As expected, numerous putative insect loci (9419 independent sequences) were retrieved from the galled sample. Fewer putative insect contigs were retrieved from the ungalled sample (671). Even samples that appear to be ungalled may have undetected eggs or young nymphs that have not yet formed visible galls, despite efforts to exclude psyllid presence from ungalled samples. The presence of insect contigs in ungalled samples may therefore reflect this undetected psyllid presence. The strong representation of insect contigs in leaf transcriptomes indicates the potential ease of co-analyzing insect and plant gene expression. When the insect contigs were blasted against the pea aphid genome a large number of putative insect gene orthologues were identified. The insect contigs are not considered further in this paper.

### Presence/absence and differential expression of plant contigs from galled and ungalled leaf of the same plant and their *Arabidopsis* gene orthologues

**Fig. 2:**
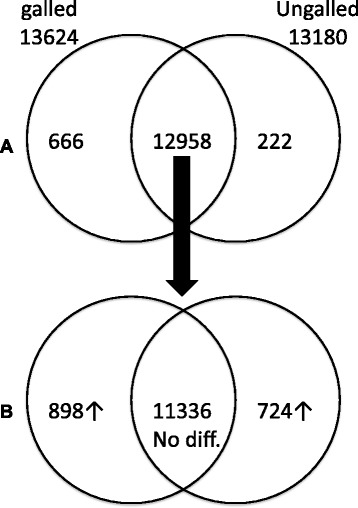
**a** Genes (*Arabidopsis* orthologues) present in galled and ungalled samples of *Metrosideros* genotype 816. 666 genes are detected in the galled sample but not in the ungalled. **b** Differential expression. Of the 12958 genes detected in both samples, 898 show substantially greater expression in the galled sample

The primary analysis used here is the comparison of galled and ungalled leaves in carefully matched samples of the same individual tree (816). Because galling causes a massive disruption to the phenotype of the leaf with implied disruption to physiology and development we were particularly interested in genes present under galling but not detectable in normal leaves, i.e. genes switched on *de novo* as a result of the galling trauma. We found 666 such *Arabidopsis* gene orthologues in the genotype 816 comparison (Fig. 2). This dataset we refer to as the tree 816 (galled only) dataset. We examined the tree 816 (galled only) dataset for auxin related genes and found a surprisingly large number of genes that are known to be expressed in response to exogenous application of auxin (Table 2), notably genes in the SAUR and GH3 gene families. We also found genes annotated to auxin response to be enriched in a GO analysis of the tree 816 (galled only) dataset (see below).

In addition to the auxin responsive genes present only in the galled sample, there are some auxin response genes that, while present in the ungalled dataset, are significantly upregulated in the galled sample. These include 6 SAUR family genes (AT3G12955.1, AT1G14000.1, AT1G10210.1, AT4G36800.1, AT5G63310.1, AT3G61900.1) and genes involved in auxin signalling, including the auxin response transcription factor ETTIN (ARF3: AT2G33860.1) and VH1-INTERACTING KINASE (VIK) which is involved in auxin and brassinosteroid signalling.

### Gall-faithful gene transcripts not present in ungalled leaves

**Fig. 3:**
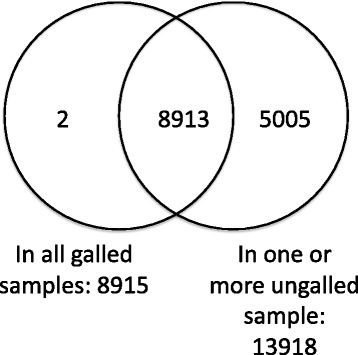
Venn diagram of genes present in all galled samples and genes present in at least one ungalled sample. Only two genes (*Arabidopsis* orthologues) have evidence of expression in all galled samples yet are absent in all ungalled samples. These genes are discussed in Results

**Fig. 4:**
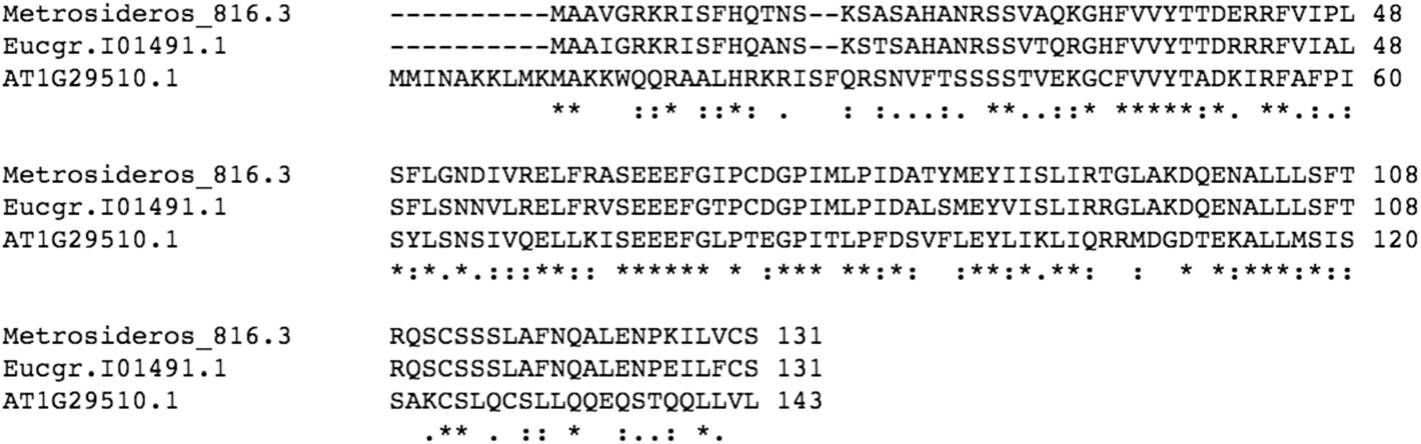
Alignment of putative SAUR67 protein from *Metrosideros* with putative *Eucalyptus* and *Arabidopsis* orthologues

The previous analyses (above) focused on genotype 816. Next, in order to test the generality of the results and to find consistent expression indicators of galling, we analysed the dataset for genes expressed in all of the diverse group of galled samples and absent in all of the ungalled samples (Table 1). Two genes were consistently recovered as transcripts from all individuals with galls yet undetectable in all samples without galls (Fig. 3). These gall-faithful and gall-exclusive genes are potentially interesting as putative markers of the galling response. One gene corresponds to one of the auxin responsive genes, SMALL AUXIN UPREGULATED 67 (SAUR67: AT1G29510), discussed above. It should be noted that SAUR67 (so named in TAIR version 10) has previously been referred to as SAUR68 [37]. Expression of several genes of the SAUR family appear to be associated with the galling response in *Metrosideros* but this member appears to be particularly consistent, not occurring in any ungalled leaves. The protein alignment of the *Metrosideros* sequence (GenBank: KT884616) with *Eucalyptus* (Eucgr.I01491) and *Arabidopsis* putative orthologs is shown in Fig. 4.

**Table 1:** *Metrosideros* leaf transcriptome sequencing

	Genotype 831 (glabrous)	Genotype 845 (glabrous)	Genotype 816 (inter-mediate)	Genotype 809 (pubescent)	Genotype 846 (glabrous)
*No galls*	*-*	*-*	*816.1*	*809*	*846.2*
**Flat galls**	**831.4**	**-**	**816.3**	**-**	**-**
**Flat and cone galls**	**-**	**845.3**	**-**	**-**	**-**
		**845.5**			

Individuals = 5; libraries sequenced = 7 (galled samples = 4 [bold]; ungalled samples = 3 [italic]). The first number indicates the genotype (e.g. 816) and the number after the point indicates the sample used for library preparation (where multiple samples were collected, e.g. 816.1, 816.3)

The other gene corresponds to the *Arabidopsis* gene HARDY (HRD). HRD (AT2G36450) is of considerable interest as it encodes a drought resistance gene [38] as a member of the dehydration responsive element binding (DREB) subfamily A-4 of the ERF/AP2 transcription factor family [39]. *Arabidopsis* HARDY has three putative paralogues in *Eucalyptus* (Eucgr.K02071.1, Eucgr.K00220.1, Eucgr.A01537.1) consistent with the expansion of DREB genes in that genus [40]. The *Metrosideros* sequence has highest similarity to Eucgr.K02071.1.

Expression of this gene in galled leaves may possibly be indicative of reduced water potential. Several other drought adaptation genes of the late embryogenesis abundant (LEA) gene family are also found in the galled leaf (816.3) but not in ungalled (816.1): AT2G18340, AT5G06760, AT2G40170, AT1G52690. LEA proteins generally accumulate in response to reduced water availability. A study of galling of the invasive plant *Parthenium hysterophorus* by *Epiblema strenuana* (Lepidoptera: Tortricidae) showed a number of physiological effects leading to reduced water potential and drought stress [41].

### Gene ontology over-representation analyses

For gene ontology enrichment analyses we used the *Arabidopsis* mappings, as the *Arabidopsis* genome has a rich functional annotation. For the genes expressed only in the galled leaf (tree 816), among the comprehensive set of Biological Process GO terms tested, 34 showed evidence of enrichment with a p-value <0.05. However only 7 of these survived false discovery rate (FDR) correction for multiple testing (Table 3). Of particular interest is “response to auxin stimulus” as auxin has been implicated in many other gall systems [5, 6]. The expression of a greater-than-expected number of auxin responsive genes under galling supports the conclusion that an auxin response is involved in the *Metrosideros* system.

In contrast, the genes present in the ungalled leaf but not in the galled leaf (222) had no significant over-representation of any biological process when FDR corrected. The genes present in both galled and ungalled leaves (12958) had a very large number of biological process GO categories significantly enriched even when corrected for multiple testing (242): as expected these were categories relating to photosynthesis and general leaf development and physiology.

To assess whether the over-representation of auxin response genes is general in galled leaves, we examined pairwise comparisons of all galled samples vs all ungalled samples using all genotypes (Table 4). Genes expressed in galled leaves but not in ungalled, were enriched in GO term “response to auxin” in seven of the pairwise comparisons. In this test we are only testing enrichment for auxin response genes so multiple-test correction is not strictly necessary. However, we note that in six of these seven cases the reslts would also be significant if all GO categories were tested. Importantly, by contrast, none of the ungalled samples showed enrichment for this GO term in any pairwise comparisons (Table 4). Although only 7 out of 12 pairwise comparisons showed significant GO enrichment for auxin respnse in galled leaves, it should be noted that all the galled samples express auxin response genes not found in ungalled leaves (e.g. SAUR67 above).

## Discussion

### An auxin response is associated with gall formation

The significant enrichment of auxin-response genes associated with gall-formation in this system is strongly indicative of phytohormone involvement in gall development. Our results show that expression of auxin response genes is general in all galled samples. This upregulation, in many samples, involves a particularly large number of auxin response genes, sufficient for a significant enrichment to be detected, in galled leaves only, in over half the pairwise comparisons between galled and ungalled leaves.

This is consistent with many previous studies, which have also shown auxin involvement [5, 6, 10, 12, 42] and auxin response gene expression [9]. By contrast we observe no obvious transcriptional activation or upregulation of auxin synthesis genes, which implies that the auxin may be exogenous, in which case it is presumably supplied by the immature insect within the gall. This accords with recent studies that have shown that diverse groups of insects can synthesize auxin [5, 7]. No study has yet determined whether psyllids synthesize auxin, but such a finding would not be surprising, and could be investigated. More puzzling is how very similar species of psyllids (such as the flat galler, *Trioza nov. sp.* 1, and the cone-galler, *Trioza nov. sp.* 2) produce such different types of gall. Not only are the gall shapes different but the galls dehisce by different mechanisms on different sides of the leaf. Auxin alone is unlikely to be the sole agent as it is difficult to see how differences in auxin production alone could produce such developmentally different outcomes in terms of gall morphology.

### The potential for co-sequencing of insect and plant transcriptomes and cross species network analyses

One potential avenue for further progress is to examine the differences in gene expression in the immature insect over different gall developmental stages, looking for (for instance) differences in gene expression between cone-gallers and flat-gallers. This would be particularly powerful if done in the context of correlated changes in plant gene expression profiles.

We have shown how insect and plant transcriptomes can be co-sequenced in the same sample and the two RNA populations separated in silico. The main problem is the lack of a well-annotated hemipteran genome. The pea aphid (*Acyrthosiphon pisum*) is the closest completely sequenced genome [43] but annotation is still progressing. Very well-annotated genomes such as the *Drosophila* genome are too phylogenetically distant to be of much direct use.

By contrast it is possible, as we have shown here, to use the *Eucalyptus* genome to characterize the *Metrosideros* transcriptome; and *Arabidopsis*, which has the best annotated plant genome, is not too distant to be cross-mapped gene-by-gene with the *Eucalyptus* genome and *Metrosideros* transcriptome. Whole genome sequencing projects are currently in progress for *Metrosideros* and there is a good prospect for the release of a *Metrosideros* whole genome assembly in the near future. There are also whole genome sequencing projects for two psyllids: the potato psyllid (*Bactericera cockerelli*) and the Asian citrus psyllid (*Diaphorina citri*). Release of these genomes, particularly if they can be well cross-annotated with the pea aphid *Acyrthosiphon pisum* genome, may facilitate the elucidation of plant-insect co-expression networks during psyllid gall formation. What has been called “the power of paired genomes” [44] could potentially lead to the elucidation of a detailed plant-insect interactome and an understanding of how two separate developmental systems co-evolve.

### The Hawaiian *Metrosideros* gall radiation as a system for gall biology

On the face of it, an endemic insect-plant system on remote islands in the Pacific may not seem a promising model for understanding a fundamental biological phenomenon. However, the Hawaian *Metrosideros*-feeding psyllids have some interesting features: they comprise a number of very closely related insects that have all evolved on the same host yet have come to interact with that host very differently. Some have reverted to a free-living lifestyle without galling, while the galling species variously produce stem-galls, flat galls and cone galls. They all manipulate the genome of the same host species to do so. It is therefore not only possible to discover how galling mechanisms work, but also how they evolve.

## Methods

### Sampling

*Metrosideros* leaf tissue was collected from five plants in a *Metrosideros* common garden situated near Volcano Village (N 19.475594, W −155.260161, 1265 m), on the island of Hawai’i (Table 1). The sampling strategy was two part. First: carefully matched leaf pairs (galled and ungalled) from the same individual plant (816) were sampled at the same time and height in the canopy (breast height: ~1.5 m above ground level). Secondly, to test the generality of the results, a further four individual plants were selected to represent a range of leaf morphologies (glabrous, intermediate, pubescent) and gall types (flat leaf and cone leaf galls) (Table 1). All samples were collected on the same day (11^th^ March 2014) between 11 am and 3 pm. All leaves were selected from the 2^nd^ or 3^rd^ node below the terminal leaf bud. Leaf tissue was flash frozen in liquid nitrogen in the field and transported in a dry shipper to the University of British Columbia, Vancouver, for sequencing.

### RNA extraction and sequencing

Leaf samples were stored at −80 C before RNA extraction. RNA was processed as previously described [45]. Briefly, RNA was extracted using Pure LinkTM Plant RNA Reagent (Invitrogen). RNA was quantitated using a QubitR 2.0 (Life Technologies) and quality-checked using an Agilent 2100 Bioanalyzer. Only samples with a RIN value of 7 or above were used for library construction. Sequencing libraries were prepared and barcoded using an Illumina Library Preparation kit. All seven libraries (Table 1) were sequenced in one lane of an Illumina Hi-Seq 2000 at the sequencing facility at the Biodiversity Research Centre, University of British Columbia (UBC), generating 100 bp paired end reads. Reads (Fastq files) for each sample are deposited in the European Nucleotide Archive (ENA).

### Processing of reads, assembly of contigs and identification of contigs to genes

The raw reads were assembled to contigs in Trinity r20140717 [46] using recommended parameters. The contigs were blasted against the complete CDS library of *Eucalyptus grandis* downloaded from Phytozome [47] using blastn [48] and an expectation value of 1e-6. Only their top high scoring *Eucalyptus* hit was then used for further analysis of the contigs. A second blast search was performed, with similar parameters, against a previously assembled transcriptome of an adult *Trioza nov. sp.* 1 psyllid, assembled in Trinity (Michael Brewer pers. comm.) in order to identify the psyllid component present in the sequenced material.

Once contigs had been matched to *Eucalyptus* genes, they were identified as their putative *Arabidopsis* orthologs by means of a pre-computed mapping table as available from Phytozome version 9: http://phytozome.jgi.doe.gov [47]. This table was used to identify orthologs between the *Eucalyptus grandis* (version 1.0) gene annotation to the *Arabidopsisthaliana* (version 10) gene annotation. Lists of transcripts (as *Eucalyptus* gene orthologs) present in each sample were constructed for comparing galled and ungalled samples (presence/absence analysis).

### Expression analysis

The main analyses in this paper were conducted with data on the presence/absence of contigs in different samples. However, quantitative differential expression analysis was also performed using Trinity [46] on specimen 816 by comparing galled (816.3) and ungalled (816.1) leaves. In order to compare read count, the raw sequencing data from both samples were pooled together. The reads were then aligned and estimated for abundance using the toolset provided in the Trinity package. From these results a minimum FPKM threshold was estimated and a measure of abundance of specific contigs per sample can then be made. Using Trinity's provided expression analysis tools, differential expression was calculated between the two leaf samples implementing the ‘edgeR’ method [46, 49]. Using the initial contig count and FPKM estimation, true FPKM values were generated and normalised. Finally, contigs were defined into associated clusters per sample in R 3.2.0 [50]. Contigs identified as being present in both samples and differentially expressed were then blasted (as per above) in order to identify *Eucalyptus* gene orthologues and ultimately *Arabidopsis* orthologues (again as above).

### Analytical methods

Sequential analysis was performed (see sampling above), first using genotype 816 (matched galled/ungalled libraries: Tables 2 and 3). Finally the generality of the results were tested using all libraries (Table 4). Genes were analyzed for gene ontology term enrichment using the AmiGO Term Enrichment Service http://amigo.geneontology.org/rte and agriGO http://bioinfo.cau.edu.cn/agriGO/ [51].

**Table 2:** Auxin responsive genes (*Arabidopsis* orthologues) present as transcripts in galled sample (816.3) and absent in ungalled sample (816.1)

*Arabidopsis* identifier	Gene name	Gene description
AT1G29500.1	SAUR66, SMALL AUXIN UPREGULATED RNA 66	SAUR-like auxin-responsive protein family
AT4G38840.1	SAUR14	SAUR-like auxin-responsive protein family
AT1G29450.1	SAUR64	SAUR-like auxin-responsive protein family
AT4G34770.1	SAUR1	SAUR-like auxin-responsive protein family
AT3G12955.1	SAUR74	SAUR-like auxin-responsive protein family
AT4G34810.1	SAUR5	SAUR-like auxin-responsive protein family
AT3G12830.1	SAUR72	SAUR-like auxin-responsive protein family
AT1G29420.1	SAUR61	SAUR-like auxin-responsive protein family
AT2G36210.1	SAUR45	SAUR-like auxin-responsive protein family
**AT1G29510.1**	**SAUR67 [SAUR68]**	**SAUR-like auxin-responsive protein family**
AT1G28130.1	GH3.17, GRETCHEN HAGEN3.17	encodes an IAA-amido synthetase
AT2G47750.1	GH3.9	encodes an IAA-amido synthetase gene
AT5G54510.1	GH3.6, DFL1, DWARF IN LIGHT 1	encodes an IAA-amido synthetase
AT2G01200.2	IAA32, INDOLE-3-ACETIC ACID INDUCIBLE 32, MEE10	belongs to auxin inducible gene family
AT1G74660.1	MIF1, MINI ZINC FINGER 1	encodes non-transcription factor zinc finger domain protein
AT1G56010.2	ANAC021, ANAC022, ARABIDOPSIS NAC DOMAIN CONTAINING PROTEIN 21, 22, NAC DOMAIN CONTAINING PROTEIN 1, NAC1	encodes a transcription factor involved in shoot meristem formation
AT2G42580.1	TETRATRICOPETIDE-REPEAT THIOREDOXIN-LIKE 3, TTL3	appears to play a role in brassinosteroid and auxin signaling
AT3G11260.1	WOX5, WUSCHEL RELATED HOMEOBOX 5	maintenance of meristem identity

SAUR67 (bold) is present in galled samples from all individuals and absent in all ungalled samples

Note: SAUR67 has previously been referred to as SAUR68 [38]

**Table 3:** Gene Ontology (GO) over-representation analysis, showing those GO categories significantly enriched (at FDR q-value <0.05) in galled leaf

Gene category	Number in *Arabidopsis* genome	Unique to galled (816.1 vs 816.3)	p-value (FDR)
All genes with annotated *A. thaliana* ortholog	37767	666	
GO:0006270 DNA replication initiation (replication initiation/All)	13 (0.0003)	6 (0.009)	0.00000059 (0.00062)
GO:0006260 DNA replication (DNA replication/All)	117 (0.0031)	11 (0.016)	0.0000027 (0.0014)
GO:0009791 post-embryonic development (Post-embryonic/All)	705 (0.018)	29 (0.043)	0.000042 (0.015)
GO:0006825 copper ion transport (copper/All)	23 (0.0006)	5 (0.0075)	0.00011 (0.027)
**GO:0009733 response to auxin stimulus (auxin/All)**	**360 (0.0095)**	**18 (0.027)**	**0.00013 (0.027)**
GO:0006259 DNA metabolic process (DNA metabolic/All)	405 (0.0107)	19 (0.028)	0.00018 (0.032)
GO:0007017 microtubule-based process (microtubule/All)	114 (0.003)	9 (0.013)	0.0003 (0.046)

Genes in GO:0009733 response to auxin stimulus (bold) are listed in Table 2

**Table 4:** Gene Ontology (GO) over-representation of category GO:0009733 “response to auxin stimulus” in all pairwise comparisons between ungalled and galled leaf

PAIRWISE COMPARISON		GO category: response to auxin	
Galled vs.	ungalled	ENRICHED IN GALLED	ENRICHED IN UNGALLED
816.3	816.1	** 0.00013 (0.027)	n.s.
816.3	809	n.s.	n.s.
816.3	846.2	**0.0000062 (0.0027)	n.s.
831.4	816.1	**0.00019 (0.046)	n.s.
831.4	809	n.s.	n.s.
831.4	846.2	n.s.	n.s.
845.3	816.1	*0.00099 (0.32)	n.s.
845.3	809	n.s.	n.s.
845.3	846.2	**0.00023 (0.0054)	n.s.
845.5	816.1	**0.000076 (0.005)	n.s.
845.5	809	n.s.	n.s.
845.5	846.2	**0.000013 (0.0012)	n.s.

The results show that significant (*p* < 0.05) enrichment only occurs in galled samples, never in ungalled. A double asterisk indicates significant enrichment that is also significant after multiple test correction (q-value given in brackets) at q < 0.05 (not strictly necessary in this case as only a single GO category was tested)

* = *p* < 0.05, ** = also significant after multiple test correction, N.S. = non-significant

### Availability of supporting data

All short read data sets used for the analyses in this article are deposited in EBI’s European Nucleotide Archive (ENA), under study number PRJEB11301, accessible at www.ebi.ac.uk/ena/data/view/PRJEB11301. The sequence of *Metrosideros* SAUR67 (full length cds) is deposited in GenBank (NCBI), accession number: KT884616 (MpSAUR67).

## Abbreviations

AP2: APETALA2 [gene]
BLASTn: Basic local alignment search tool (nucleotide)
bp: Base pairs
CDS: Coding DNA sequence
DREB: Dehydration responsive element binding [gene family]
EBI: European Bioinformatics Institute
ENA: European Nucleotide Archive
ERF: Ethylene-responsive element binding factor [gene family]
FDR: False discovery rate
FPKM: Fragments Per Kilobase of transcript per Million mapped reads
GH3: Gretchen Hagen3 [gene family]
GO: Gene ontology
HRD: HARDY [gene]
IAA: Indole-3-acetic acid
LEA: Late embryogenesis abundant [gene family]
*M. polymorpha*: *Metrosideros polymorpha*
NCBI: National Center for Biotechnology Information
nov.sp.: New species
RIN: RNA integrity number
RNA: Ribonucleic acid
SAUR: Small auxin upregulated [gene family]
UBC: University of British Columbia
